# Myometrial contractility influences oxytocin receptor (OXTR) expression in term trophoblast cells obtained from the maternal surface of the human placenta

**DOI:** 10.1186/s12884-015-0656-3

**Published:** 2015-09-16

**Authors:** Dariusz Szukiewicz, Anna Bilska, Tarun Kumar Mittal, Aleksandra Stangret, Jaroslaw Wejman, Grzegorz Szewczyk, Michal Pyzlak, Jacek Zamlynski

**Affiliations:** Department of General & Experimental Pathology with Centre for Preclinical Research and Technology (CEPT), Medical University of Warsaw, ul. Pawinskiego 3C, 02-106 Warsaw, Poland; Department of Obstetrics & Gynecology, Second Faculty of Medicine, Medical University of Warsaw, ul. Kondratowicza 8, 03-242 Warsaw, Poland; Department of Pathology, Professor Witold Orlowski Public Clinical Hospital, Medical Center for Postgraduate Education, Czerniakowska 231, 00-416 Warsaw, Poland; Gynecology Clinical Care Unit, Department of Obstetrics and Gynecologic Oncology in Bytom, Medical University of Silesia, ul. Batorego 15, 41-902 Bytom, Poland

**Keywords:** Oxytocin, Oxytocin receptor, Trophoblast, Human placenta, Myometrial contractility, Uterine contractions

## Abstract

**Background:**

Oxytocin (OXT) acts through its specific receptor (OXTR) and increased density of OXTR and/or augmented sensitivity to OXT were postulated as prerequisites of normal onset of labor. Expression of OXTR in the placental term trophoblast cells has not yet been analyzed in the context of contractile activity of the uterus. Here we examine comparatively OXT contents in the placental tissue adjacent to the uterine wall and expressions of OXTR in this tissue and corresponding isolated placental trophoblast cells.

**Methods:**

Twenty eight placentae after normal labors at term (group I, *N* = 14) and after cesarean sections performed without uterine contractile activity (group II, *N* = 14) have been collected. Tissue excised from the maternal surface of examined placenta was used for OXT concentration measurement, cytotrophoblast cell cultures preparation and immunohistochemistry of OXTR. Concentration of OXT was estimated in the tissue homogenates by an enzyme immunoassay with colorimetric detection. Cytotrophoblast cells were isolated using Kliman’s method based on trypsin, DNase, and a 5–70 % Percoll gradient centrifugation. The cultures were incubated for 5 days in normoxia. Both placental specimens and terminated cytotrophoblast cultures were fixed and embedded in paraffin before being immunostained for OXTR. Using light microscopy with computed morphometry for quantitative analysis, OXTR expressions were estimated in calibrated areas of the paraffin sections.

**Results:**

There were not significant differences between the groups in respect to the mean OXT concentration. However, in both groups the median value of OXT concentration was significantly (*p* < 0.05) higher in the tissue obtained from the peripheral regions of the maternal surface of the placenta, compared to the samples from the central region of this surface. In placental tissue the mean expression of OXTR in group I was significantly (*p* < 0.05) increased by approximately 3.2-fold and 3.45-fold (the samples collected from central and peripheral regions, respectively) compared to the values obtained in group II.

In the isolated primary trophoblast cultures the differences were even more evident (*p* < 0.02) and the mean change in OXTR expression in group I comprised approximately 6.9-fold increase and 6.5-fold increase (the samples collected from central and peripheral regions, respectively) compared to the values obtained in group II.

**Conclusions:**

Upregulation of OXTR within placental trophoblast cells localized close or adherent to uterine wall may play a crucial role in labor with efficient contractile activity (vaginal delivery). Further studies may disclose if this local OXT/OXTR signaling is utilized in the third stage of labor to elicit placental detachment or contribute in a more versatile way throughout the labor period.

## Background

Normal onset of labor with maintained regular intrauterine contractions leading to shortening and cervical dilation (first stage), expulsion of the fetus (second stage) and placental delivery followed by uterine contraction and retraction (third stage) are needed for successful pregnancy outcome [1]. Some mechanism that trigger the contractile activity of the myometrium are still not clearly understood, including those responsible for detachment of the placenta [2]. Local interactions between the two adjacent surfaces: placental (the maternal side) and the corresponding decidual/myometrial surface area would be considered extremely significant in terms of ability to maintain pregnancy to term and the proper course of parturition [3].

Interestingly, the role of oxytocin (OXT) seems to be more complex than it was suggested before. This nonapeptide (or more precisely the octapeptide amide with amino acid sequence: Cys-Tyr-Ile-Gln-Asn-Cys-Pro-Leu-Gly-NH_2_) was discovered initially as a neuropeptide [4, 5]. In the hypothalamus, OXT is synthesized as an inactive precursor protein that is progressively activated by gradual hydrolysis into smaller fragments while it is being relocated along the axon towards the posterior pituitary. The last step of this activation is catalyzed by peptidylglycine alpha-amidating monooxygenase (PAM, EC 1.14.17.3) [6].

Finally, OXT is released to the blood stream and acts directly via axon terminals on OXT receptors (OXTR) in the CNS (e.g. the nucleus accumbens) [7]. Amongst other peptides that act locally (e.g. corticotropin-releasing hormone, and dynorphin), OXT and vasopressin are the only known hormones secreted by the human neurohypophysis to act at a distance [8].

The structure of OXT is higly conserved in placental mammals. However, in some new world primates, a novel structures of OXT have been reported [9]. OXT, and another very similar (differs by only two amino acids) nonapeptide – vasopresin, were isolated and synthetized in 1953 by Vincent du Vigneaud [10, 11]. OXT has a disulfide bond between the two cysteines, and reduction of the disulfide bond inactivates OXT [5].

An intricate interplay between OXT and OXTR is responsible for various functions of OXT acting as neuroregulator or in a paracrine manner in ovary or uterus, including materno-placento-fetal unit [1, 6]. Despite of the fact that OXT evokes uterine contractions, some discrepancies about the exact role of OXT in parturition have arisen in the mid 1980s, after the first suggestion that OXT synthesis also occurs in peripheral tissues [12, 13]. Outside the brain, synthesis of mRNA encoding OXT was demonstrated in many human tissues, including intrauterine tissues in advanced gestation, principally in the decidua but also in the placental membranes (chorion, amnion) [14, 15]. These findings may lead to suggestion that paracrine or autocrine role rather than, or in addition to, an endocrine role of OXT should be expected in respect of contractile activity of the human uterus [16, 17]. So if OXT is not considered solely as an endorine factor, many previous conclusions about its effect on the course of subsequent stages of pregnancy and labor should be retested [6].

In contrast to vasopresin, OXT is known to have only one type of specific, high-affinity receptor (OXTR). The human OXTR mRNAs were found to be of two sizes, 3.6 kb in breast and 4.4 kb in ovary, endometrium, and myometrium. The OXTR gene is present in single copy in the human genome and was mapped to the gene locus 3p25–3p26.2 [18, 19] OXTR belongs to the rhodopsin-type (class I) G protein (Gαq11)-coupled receptor family that requires Mg^2+^ and cholesterol [20]. OXTR is coupled to phospholipase C (PLC, EC 3.1.4.11) which controls the generation of diacylglycerol (DAG) and inositol 1,4,5 – trisphosphate (InsP3). Thus, after activation of OXTR, inositol triphosphate triggers an increase in Ca^2+^ influx from both extracellular and intracellular stores, whereas DAG stimulates activation of protein kinases type C (PKC, EC 2.7.11.13), which phosphorylates unidentified target proteins. The OXTR has a weak ligand selectivity profile. It was proved that OXTR is able to couple to different G proteins, including G_i_ and G_s_ proteins [21].

Expression of OXTR in the pregnant and non-pregnant myometrium differs significantly [22, 23]. Activation of functional OXTRs stimulates cell proliferation in human placental trophoblast and choriocarcinoma cell lines [24]. Increased density of OXTR and/or augmented sensitivity to OXT were postulated as prerequisites of effective parturition with normal induction of uterine contractions [22, 25]. These changes observed at transcription and protein levels during pregnancy resulting in heterogenous OXTR expression in the term myometrium [22].

However, there are still serious controversies about exact role of OXTR in the initiation of uterine contractions in term pregnancy. It is not known precisely how the increase in OXTR mRNA concentrations in the myometrium and decidua is regulated and what is the contribution of OXTR within the placental tissue. Both, existence of a desensitization mechanism for post-transcriptional regulation of the OXTR, and interplay between OXT and prostaglandins (PGF_2α_, PGE) may be rather responsible for the crucial stage of the liberation of uterine contractions at term [26, 27]. Quite recently the new approach has arisen suggesting that OXT and OXTR interactions are crucial to preserve myocyte regeneration and proliferation rather than induction of the uterine contractile activity [28].

Expression of OXTR in the placental term trophoblast cells of the maternal side, contiguous to the uterine wall has not yet been analyzed thoroughly in humans in the context of contractile activity of the uterus. Development of a suitable model for investigation of OXTR expression in the cytotrophoblast enabling a novel approach to this issue may yield interesting contribution to our understanding of the third stage of labor, above all else may shed some light on the mechanism of placental detachment. The interactions between placental prostaglandins and OXT, for example, have been discussed comprehensively, however, not in relation to trophoblast pool of OXTR [29].

The aim of this study was to examine comparatively OXT contents in the placental tissue adjacent to the uterine wall and expressions of OXTR in this tissue and corresponding isolated placental trophoblast cells obtained after normal labors at term and after cesarean sections performed without spontaneously induced uterine contractions (group I and II, respectively).

## Methods

The study was conducted in compliance with international and local laws of human experimentation and was officially approved by local ethics committee of the Medical University of Warsaw (ethical clearance approval number: KB21/2011). Each woman has signed a written consent for the use of her placentae as the source of trophoblast cells. Strictly speaking, this work has been carried out in accordance with The Code of Ethics of the World Medical Association (Declaration of Helsinki) for experiments involving humans, and the Uniform Requirements for manuscripts submitted to Biomedical Journals have been fulfilled.

### Placental tissue collection

Fourteen placentae obtained from nulliparas after natural unifetal vaginal births (group I) were compared with equal number of gestationally matched placentae collected after elective cesarean sections (group II). The indications for cesarean section were high-grade myopia in pregnant woman and breech presentation of the fetus. More detailed clinical characteristics of the two homogenous (except for the mode of delivery) groups are given in Table 1. As previously mentioned, uterine contractile activity was not observed in group II.

**Table 1:** Clinical characteristics of the two groups studied (median and range). The groups may be treated as homogenous, except for the mode of delivery

Parameter	Group I (vaginal delivery)	Group II (cesarean section)
Number of patients/newborns/placentas/specimens obtained (*N*)	14/14/14/42	14/14/14/42
Parity	0	0
Gestational age in days (median; range)	283; 269–290	276; 265–282
Preterm uterine contractions	Not reported	Not reported
Contractile activity of the uterus at time of delivery	Present and effective	Not present
Oxytocin treatment in the first and second stage of labour	Not used	Not used
Blood pressure during pregnancy	All records within normal range^a^	All records within normal range^a^
Proteinuria during pregnancy	Not present	Not present
Liver blood tests (aminotransferases enzymes, AST and ALT levels)	Within normal range^b^	Within normal range^b^
Diabetes during pregnancy	Not present	Not present
Body mass index <21 or >35	None	None
Mother’s blood (III trimester): hematocrit (Ht), hemoglobin (Hb), red blood cell (RBC) count, mean cell hemoglobin concentration (MCHC)	All within normal ranges^c^	All within normal ranges^c^
Other identified risk factors	None	None
Birth weight in grams (median; range)	3380; 2990–3730	3190; 2850–3860
5-minute Apgar score below 8 points^d^	None	None
Sex of newborns (M, male; F, female)	7 M + 7 F	8 M + 6 F
Weight of placenta in grams (median; range)	660; 559–790	650; 550–810

^a^The normal range of blood pressure was defined as systolic pressure between 100 and 140 mmHg, and diastolic pressure between 60 and 90 mmHg

^b^The normal range of values for AST is 5–40 units per liter of serum and the normal range of values for ALT is 7–56 units per liter of serum

^c^Hb levels 10.0–13.5 g/dl, RBC count 3.2–4.4 million/μl, MCHC 319–355 g/L, Ht 31–41 %

^d^Apgar score is determined by evaluation newborn on five criteria that form a backronym (**A**ppearance, **P**ulse, **G**rimace, **A**ctivity, **R**espiration) on scale from “0“ to “2“. The scores are added up and the total sum is an Apgar score. Scores 7 and above are generally nomal, 4–6 fairly low, and 3 and below generally regarded as critically low

**Fig. 1:**
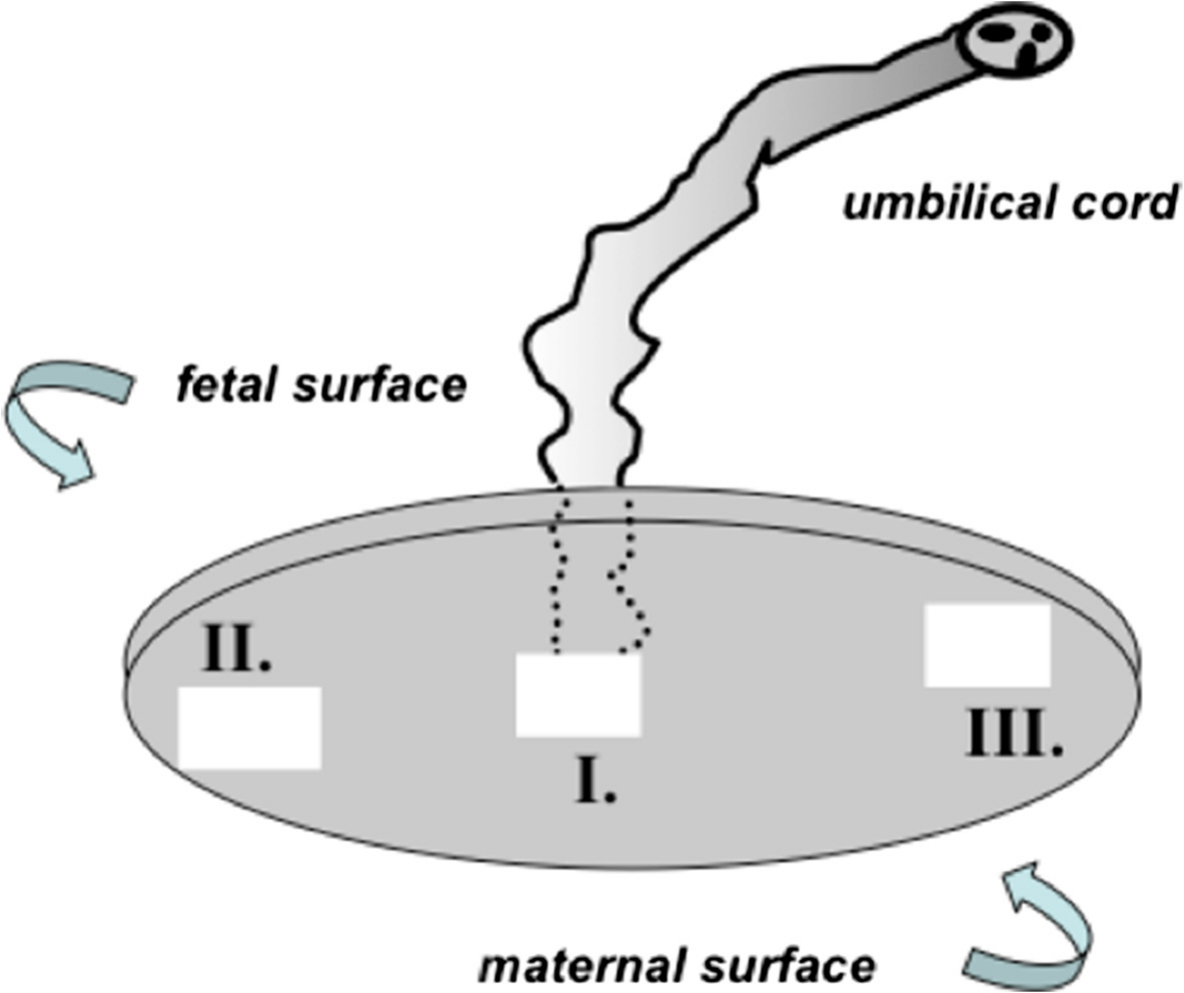
Location of samples (I – III) collected from the maternal surface of the placenta. The mean weight of the sample: 49.76 ± 4.54 g

From each collected placenta, three specimens were excised in a standardized manner from the region contiguous to the maternal surface of the placenta: the first one – from the central part, the next two – from peripheral regions of the placental maternal surface (Fig. 1). All placentae used in this study were complete and characterized by a central umbilical cord insertion. In all cases enrolled in group I, expulsion of the placenta was preceded by its normal separation (so-called shiny Schultz) [30]. The management of the third stage of labor was active with umbilical cord clamped immediately after delivery of the newborn and oxytocin (5 IU) administered i.v. to the mother. Similarly, oxytocin (5 IU) was administered during cesarean section following delivery of the newborn. The tissue material obtained by this procedure was subjected either to freezing in carbon dioxide snow for OXT concentration measurement or served as a source of cytotrophoblast cells for the development of in vitro cultures. Appropriate specimens were also embedded in paraffin and used for for immunohistochemistry of OXTR.

### Measurement of OXT content

Concentration of OXT was estimated in the frozen placental excisions. During this procedure, the tissue was kept on ice. Before obtaining a lysate, the material was initially cut into about 1 mm^3^ cubes by using a razor blade on a glass plate held on ice. To perform a gentle cell disruption, the cubes were then transferred into a hand-held Potter S homogenizer from Sartorius™ Stedim Biotech GmbH. The liquefied tissue was poured into the 1.5 mL tubes and centrifuged at 10,000 xg for 3 min at 4 °C. The clear supernatant was subjected to in vitro quantitative measurement of OXT in the placental tissue homogenates by an enzyme immunoassay with colorimetric detection. The Oxytocin ELISA-Kit – (Cat. no. ADI-901-153A-0001) – Enzo Life Sciences, Inc. (USA) was applied with declared by manufacturer sensitivity level at 15 pg/ml (detection range: 15.6–1.000 pg/ml). This species independent kit uses a polyclonal antibody to OXT to bind, in a competitive manner, the OXT in the standard or sample or an alkaline phosphatase molecule which has OXT covalently attached to it. Following simultaneous incubation at 4 °C, the excess reagents are washed away and subtrate is added. After a short incubation time the enzyme reaction is stopped and the yellow color generated read on a microplate reader (Biochrom Asys UVM-340 Microplate Reader by Biochrom Ltd., Cambridge, UK) at 405 nm. The intensity of the bound yellow color was inversely proportional to the concentration of OXT in either standards or samples. Thus, the measured optical density was used to calculate the concentration of OXT. Relatively low (not exceeding 7–7.5 %) cross reactivity with Arg^8^-vasotocin (vasopressin) and very low reactivity for other related molecules (<0.02 %) provided confidence in OXT assay results. Finally, the concentration of OXT measured by ELISA were converted into ng/g of wet placental tissue. The intra-assay precision (% CV) amounted to 11.4 and the inter-assay variance (% CV) was 16.1.

### Trophoblast in vitro cultures

Concurrently with the measurement of OXT content, preparation of the cell cultures has begun. Cytotrophoblast cells were isolated using modified Kliman’s method as described elsewhere [31–33]. This method is based on trypsin, DNase, and a 5–70 % Percoll gradient centrifugation. Briefly, immediately after obtaining each specimen of the placental tissue was denuded from decidua fragments and abundantly rinsed with 0.9 % saline to remove all blood. A minced villous tissue was subjected to 30 min enzymatic digestion at 37 °C during incubation in shaking water bath in CMF-HBSS (calcium- and magnesium-free Hank’s balanced salt solution; 10 × stock solution from Life Technologies Inc. Gibco/Brl, NY, USA) with 0.125 % trypsin (Sigma-Aldrich Inc., USA; T8003) and 0.2 mg/ml deoxyribonuclease I (DNase I Type IV; Sigma; D5025). Such digestion was performed sequentially four times. Each time a freshly prepared digestive mixture (solution) was used to the next placental samples. The cells that were released at every 30 min step were pooled and filtered through two layers of gauze, and trypsin was inactivated with fetal calf serum (FCS). The filtrates were centrifuged, and the cell pellets were resuspended in PBS.

The resultant cell suspension in supernatant was filtered and fractionated on a 5–70 % Percoll (Sigma; P1644) gradient centrifugation (1200 × g for 25 min). The cell layers incorporated into a range 40-50 % (density 1.048–1.062 g/ml) have been collected, washed and resuspended (1 × 10^6^ cells/ml) in Ham’s F12/Dulbeco’s modified Eagle’s medium (1:1; Sigma; 51445C) with 15 % fetal bovine serum (Gibco/Brl; 26140–079), 1 mmol/l sodium pyruvate, 2 mmol/l L-glutamine, and 50 μg/ml gentamycin. The cultures were then incubated for 5 days under normoxic (20 % O_2_) conditions at 37 °C with 5 % of CO_2_, with the media changed daily. The purity of the cytotrophoblast culture developed from each placenta was evaluated by immunoperoxidase staining with antibodies against vimentin. Described procedure yielded preparation of a highly purified cytotrophoblast (approx. 95 % pure) with the mean viability ≥ 90 %.

### Immunohistochemistry of OXTR

#### Placental tissue samples

Human placenta paraffin 5 μm sections were subjected to the immunohistochemical staining. Goat anti-human polyclonal IgG (N-terminal) antibody to OXTR (Abnova Corp. Taipei, Taiwan; ABIN1049194; concentration 9 μg/ml) was applied as primary and chicken anti-goat IgG (heavy and light chains) as biotinylated secondary antibody (Abnova; PAB10566; 0.5 % v/v). In order to highlight the specifically bound primary antibodies to OXTR, the StreptAB-Complex/HRP Duet (Dako Cytomation, Glostrup, Denmark; K0492) was used, following the protocol recommended by the manufacturer, with 3,3’– diaminobenzidine that served as a chromogen. The appropriate negative controls for all immunostainings were established simultaneously by replacement of the polyclonal primary antibody by normal goat preimmune IgG diluted with phosphate buffered saline (PBS), containing 3 % bovine serum albumin at the same protein concentration as that used for the primary antibody.

In view of the fact that OXTR are expressed in vascular endothelial cells, it was presumed that the accuracy of OXTR expression measurement may be significantly influenced by the local differences in density of placental microvessels. To avoid this discrepancy in the results, identification of the vasculature elements in placental sections was performed using endothelial cell maker, rabbit polyclonal antibody anti-CD31 (dilution 1 : 50, Abcam Inc., Cambridge, MA, USA; ab28364). Next, a biotinylated goat anti-rabbit antibody was used as the secondary (Abcam).

Using light microscopy with computed morphometry for quantitative analysis (Quantimet 500C+ image analysis workstation provided by Leica, UK), the vascular/extravascular tissular index (V/EVTI) was estimated in calibrated areas of the placental sections, as described in detail elsewhere [34–36]. Briefly, each specimen (paraffin section) underwent three area analyses repeated by two experienced, independent observers. The single area measured with the picture analyzer amounted to 685254 μm^2^ and the total number of specimens 42 per group. The picture analysis procedure consisted in a measurement of the total vascular area. Consequently, the total lumen area of all types of identified vessels was summed up in both groups. With the purpose of a minimizing disruption caused by technical errors, especially unaxial section of the vessel, the lowest value of Ferret’s diameter was accepted as the diameter of single lumen. Thus, V/EVTI represents the ratio, which reflects intensity of vascularization and is most closely correlated with the mean density of placental microvessels.

#### Cytotrophoblast

After 5 days the cultures were terminated, formalin-fixed and paraffin-embedded. The paraffin blocks containing cytotrophoblast cells were cut in 4 μm-thick sections on a microtome with a disposable blade. Visualization of OXTR was performed using standard immunohistochemical pocedures as already described above (see Chapter 2.4.1.) with exception for V/EVTI.

#### Expression of OXTR

In order to assess OXTR expression, a quantitative immunohistochemistry based on morphometric software (Quantimet 500C+) was performed twice by two independent researchers. Next, the average values were uploaded into the result recording tab. Intensity of immunostaining was evaluated using mean color saturation parameter and thresholdings in grey-level histograms. Thus, expression of OXTR corresponded to the total immunostained area of examined sections, where the color saturation comprises segmentation/separation criteria for objects. A single analysed image area was 131574 μm^2^ (magnification ×200). In total, 126 visual fields have been analysed (9 visual fields per placenta) in each studied group.

During comparative measurements of OXTR expression in placental tissue samples in both groups, the vascular density-matched samples were analyzed. In any case, the difference between the mean V/EVTI values not exceeded ± 5 % [37]. Morphometric results comprising 90 % confidence intervals were reported as the mean fold changes. Naturally, expression of OXTR in the isolated cells of placental cytotrophoblast was estimated without taking into consideration the vascular density.

**Fig. 2:**
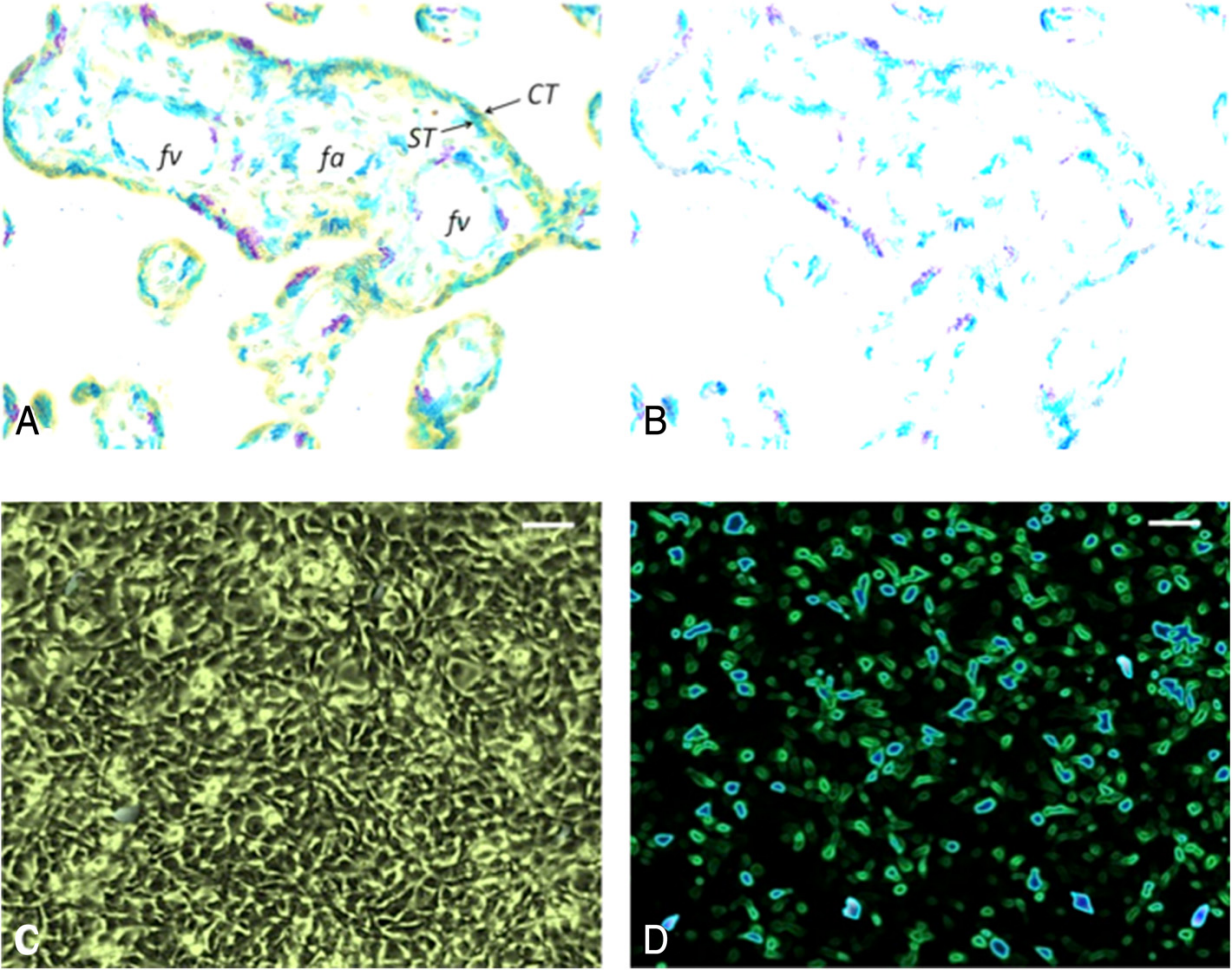
Immunohistochemical visualization of OXTR. **a**, **b** – placental samples (the image captured through optical microscope was subjected to series of digital transformations for morphometric purposes; initial magnification 400×); **c** – trophoblast cells in culture (*phase –contrast image*); **d** – trophoblast culture immunostained for OXTR (*the image digitally transformed for morphometric purposes*). The OXTR is represented by the category of blue hues ranged from dark blue to violet blue. Scale bar = 100 μm. CT – cytotrophoblast, ST – syncytiotrophoblast, fv – fetal vein, fa – fetal artery

Application of highly specific antibodies for immunostaining of OXTR together with quantitative imunohistochemistry made it possible to disclose the nature of interrelationships between contractile activity of the uterus and placental expression of OXTR, including isolated trophoblast cells (Fig. 2).

### Statistical analysis

All statistical analyses were performed using Statistica 8.0 software (Stat-Soft, Poland). Mann-Whitney’s U test was applied. The results are expressed as the mean ± SEM, medians or mean percentage values ± SEM. The differences between groups I and II (natural vaginal birth vs. cesarean section without contractile activity of the uterus) were deemed statistically significant if *p* < 0.05.

## Results

**Fig. 3:**
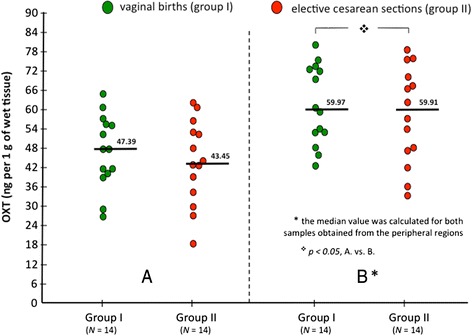
Oxytocin (OXT) concentration in placental tissue obtained from central **a** and peripheral **b** regions of the maternal surface. The median values for the groups were marked

To compare OXT levels in the placenta from vaginal ans CS births, placental extracts were analyzed by a standard ELISA assay. The results pertaining to OXT content in the placental tissue homogenates are summarized in Fig. 3. There were not significant differences between the groups in respect to the mean OXT concentration in the placental samples obtained from the maternal surface of the placenta. The mean value for the total number of samples in group I amounted to 56.82 ± 4.78 ng/g of wet tissue ± SEM, while in group II the mean OXT content was estimated to be 54.35 ± 4.93 ng/g of wet tissue ± SEM.

It is worth noting that the mean OXT concentration revealed correlation with the location of samples. However, considering that the data are not normally distributed within the groups, the median values (the middle values) have been juxtaposed.

As this may be quite important, in both groups the median value of OXT concentration was significantly (*p* < 0.05) higher in the tissue obtained from the peripheral regions of the maternal surface (Fig. 3b), compared to the samples from the central region (Fig. 3a). In group I (vaginal delivery) the respective median values amounted to 59.97 ng/g of wet tissue and 47.39, respectively, while in group II (elective cesarean section) the median values of OXT concentration were estimated to be 59.91, and 43.45 (peripheral and central region, respectively).

**Fig. 4:**
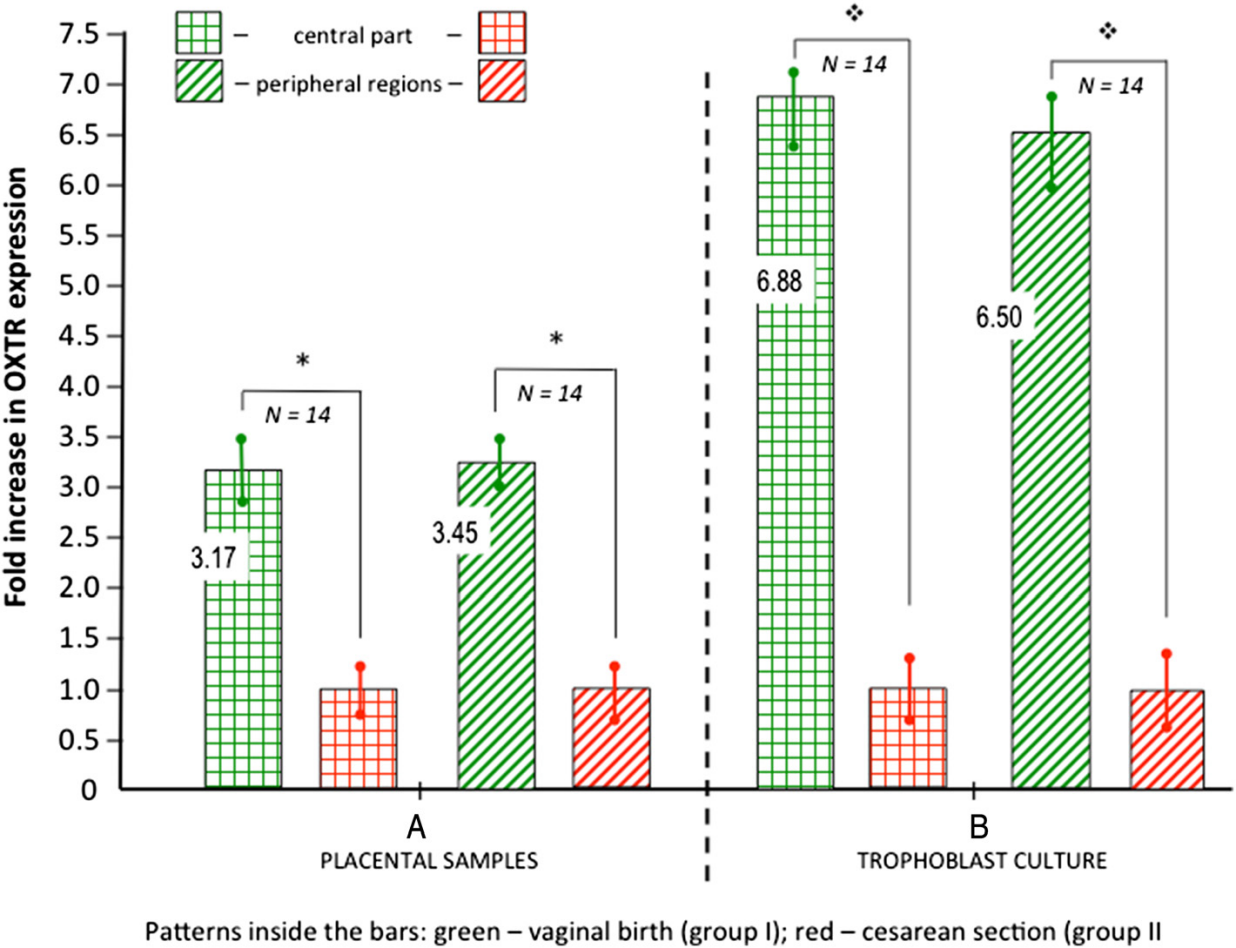
Group I vs. group II. Mean fold increase in OXTR expression in placental samples **a** and the trophoblast cultures **b**. The data are expressed as mean ± SEM (* indicates *p* < 0.05; indicates *p* < 0.02)

Relative changes in the mean OXTR expression in the placental samples and in the trophoblast cultures are shown in Fig. 4 (4a and 4b, respectively). It is clearly visible that in placental samples obtained after vaginal delivery (group I) the mean density of OXTR undergoes significant (*p* < 0.05) upregulation. The mean expression of OXTR in group I was increased by approximately 3.2-fold and 3.45-fold (the samples collected from central and peripheral regions, respectively) compared to the values obtained in group II (cesarean section).

The results of comparative evaluation of the mean OXTR expression in the isolated primary trophoblast cultures (Fig. 4b), which means that the influence of the placental vasculature was eliminated, revealed generally the same characteristics of dependencies as already observed for placental samples. However, the differences were even more evident (*p* < 0.02). The mean change in OXTR expression in group I (vaginal delivery) comprised approximately 6.9-fold increase and 6.5-fold increase (the samples collected from central and peripheral regions, respectively) compared to the values obtained in group II (cesarean section).

## Discussion

The control of timing of labor is achieved by complex interactions between mother, fetus and the placenta. Since the contact area of the maternal side of the human placenta is relatively large, this organ may influence uterine contractility throughout a pregnancy. Both local (limited to the site of placental attachment) and all-encompassing interrelationships should be better elucidated [38]. Our results indicate that despite of similar OXT concentration in the placental tissue, the OXTR density may determine onset of the myometrial contractions at the time of delivery. Independent authors already reported that the progress of labor is not related to an increase in OXT concentration, uterine contractions are not associated with changes in plasma OXT concentration and hypocontractile labor does not appear to be the result of a deficit of OXT [39]. These authors concluded that their results do not support a role of OXT during spontaneous labor unless uterine activity is controlled by extremely low plasma hormone concentrations or the uterus becomes sensitive to a constant OXT concentrations [39]. Considering our findings, the latter condition may be caused by region-specific upregulation of OXTR.

The mechanisms utilized to initiate and coordinate contractile activity of the uterus during labor are not known [1, 40]. Existence of an pacemaker, a site where the action potential (AP) is generated, forms the basis of the canonical AP propagation theory [41]. In all excitable tissues, including uterine smooth muscle cells, AP resulting from the depolarization of the cell membrane (sarcolemma) is the trigger for many intracellular events. First of all, depolarized plasma membrane opens voltage-sensitive ion channels resulting in highly increased entry of extracellular Ca^2+^ which in turn causes calcium-dependent uterine contractions, especially in calcium-sensitized myometrium [42]. Two types of APs have been observed in myometrial smooth muscle cells: simple AP with depolarization followed by rapid repolarization, and complex AP, where initial depolarization devolves into sustained plateau. It has been suggested, that these different patterns of electrical activity may result from other combinations of ionic currents during the course of given type AP [43, 44].

Apart from this, after many years of investigations, the concept of pacemaker is still an open debate since there is no evidence for the presence of cells with such histologic or electrophysiologic characteristics. It was even suggested that all uterine smooth muscle cells have the capacity to act as pacemaker cells, and thereby initiate the contractions [41]. Some researchers have postulated the existence of a unique, subendometrial junctional zone of the myometrium that may generate and regulate these bioelectrical activities [45]. Thus, changed properties of placental cells adjacent to the decidua may significantly affect contractile status of the uterus [46, 47]. As in the uterus, OXT also stimulates production of prostaglandins, including PGF_2α_ in the decidua, amnion and cytotrophoblast [48]. It was suggested that estrogen stimulated, local production of OXT within the chorio-decidual and trophoblast cells may contribute to the modulation of AP promoting formation of the complex APs [49].

Calcium sensitization (CS) is considered as a potentially significant functional mechanism for the regulation of uterine contractility. In this condition, especially after stimulation with an agonist such as OXT, a given influx of Ca^2+^ will cause a larger than expected strenght of contraction [48]. Amongst various uterotonins evaluated for their ability to evoke CS, the effects of OXT have been analyzed thoroughly. It has became evident that CS plays important role in potentialization of the contractile effects of OXT on the human myometrium [22, 49]. Our results thus suggest that, depending on OXTR expression, the same level of CS produced by similar concentrations of OXT may be sufficient or unable to generate a sustained regular uterine contractions. It should be considered whether local functional changes in placental tissue during the short period immediately preceding onset of labor may upregulate OXTR [50]. Because OXT in uterine tissue activates OXTR and another member of the G protein-coupled receptor (GPCR) family, vasopressin V_1A_ receptor, the local expression of this latter may also influence uterine contractility. However, V_1A_ receptor affinity and potency of OXT are much lower than those of vasopressin [51].

Immunohistochemical stainings of the placental samples have revealed that OXTR are expressed in both vascular endothelial cells and trophoblast cells (Fig. 3a). In view of this, assessment of OXTR expression performed in placental sections in conjunction with the mean density of placental microvessels (V/EVTI) as well as the use of isolated trophoblast cells in culture are justified in our study. In that way, we were able to demonstrate that perinatal upregulation of OXTR take place predominantly or is even limited to the trophoblast cells located on the maternal side of term placenta. To the best of our knowledge, this is the first publication pertaining to humans aimed at assessing placental OXT concentration in the context of uterine contractility and OXTR density in both placental tissue and isolated trophoblast cells.

Interestingly, in both groups the mean OXT concentration was higher in the peripheral regions of the placenta compared to the central region. It may indicate that significant differences in the basal level of CS could be an important factor influencing contractility of the uterus, possibly during delivery of the placenta. The most commonly observed birth of the placenta by the Schultz Mechanism include the formation of a retroplacental hematoma between the separating placenta and remaining spongiosa layer of the decidua. It facilitates the completion of placental separation [52]. Thus, together with an optimal expression of OXTR, different CS of the myometrium within the placental site evoked by changes in OXT concentration may play a role in the typical mechanism of placental separation during the third stage of labor. It can be speculated that in less common the Matthew Duncan mechanism of placental detachment, as well as in many disorders of the placental stage of labor, expression patterns of OXTR may be different [2, 53].

Contrary to above deduction, observed changes in the mean OXT concentration between central and peripheral regions of the maternal surface may be a consequence of dissimilar number of cells in the placental tissue obtained from different locations. This possibility was mentioned by some independent authors [54, 55]. In addition, to some extent the differences in distribution of placental microvessels and resulted diverse blood supply may contribute to the above results. It should also be taken into account that non-contemporaneous differentiation of the trophoblast in the primary cultures may influence the results pertaining to OXTR expression. The assessment of trophoblast differentiation was not performed in this study.

If we focus on the tissue level, AP propagation theory is sufficient to explain induction of the uterine contractions. However, given the difficulty AP propagation alone has explaining organ-level function, largely forgotten idea of mechanotransduction by pressure-tension sensing should be re-considered [40, 56]. According to this concept, based on Laplace’s Law, the contractions may be controlled by stretch of the uterine wall while significant increase of intrauterine pressure causes increases of wall tension. Next, the optimal level of the wall tension activates contractions throughout the uterus. It is very likely, that both AP propagation from uterine pacemaker and mechanotransduction are complementary and may be helpful with blanket conceptualization of the uterine contraction phenomenon [57]. It is possible that the mechanism of placental detachment from not initially contracting part of the uterine utilizes the pacemaker as the main mechanism of AP activation, while mechanotransduction mechanism is needed for an organ-level coordination of myometrial repetitive, synchronous contractions during normal human labor [58]. Based on our results, we suspect that interaction between OXT and OXTR is an important part of this functional machinery that may be explained using both action potential (AP) and mechanotransduction theories.

In conclusion, the results indicate that upregulation of OXTR within placental trophoblast cells localized close or adherent to uterine wall may play a crucial role in the mechanisms involved in labor with efficient contractile activity. Further studies are needed to disclose if this local OXT/OXTR signaling is utilized in the third stage of labor to elicit placental detachment or contribute in a more versatile way throughout the labor period, including initiation of labor.

## Abbreviations

AP: Action potential
CMF-HBSS: Calcium- and magnesium-free Hank’s balanced salt solution
DAG: Diacylglycerol
CNS: Central nervous system
CS: Calcium sensitization
FCS: Fetal calf serum
GPCR: G protein-coupled receptor
InsP3: Inositol 1,4,5 – triphosphate
OXT: Oxytocin
OXTR: Oxytocin receptor
PAM: Peptidylglycine alpha-amidating monooxygenase
PBS: Phosphate buffered saline
PGE: Prostaglandin E
PGF_2α_: Prostaglandin F_2α_
PKC: Protein kinases type C
PLC: Phospholipase C
V/EVTI: Vascular/extravascular tissular index
